# Sustainability trends and gaps in the textile, apparel and fashion industries

**DOI:** 10.1007/s10668-022-02887-2

**Published:** 2023-02-10

**Authors:** Stefano Abbate, Piera Centobelli, Roberto Cerchione, Simon Peter Nadeem, Emanuela Riccio

**Affiliations:** 1 Department of Industrial Engineering, University of Naples Federico II, P.Le Tecchio 80, 80125 Naples, Italy; 2 Department of Industrial Engineering, University of Naples Parthenope, Centro Direzionale Di Napoli, Isola C4, 80143 Naples, Italy; 3 Centre for Supply Chain Improvement, University of Derby, Kedleston Road, Derby, DE221GB UK

**Keywords:** Circular economy, Clothing, Fashion industry, Literature review, Network analysis, Sustainable development

## Abstract

Textile, apparel, and fashion (TAF) industries contribute significantly to global environmental pollution at every point of the supply chain. Clothing manufacturing and transportation produce a large volume of waste and high greenhouse gas emissions, often taking advantage of cheap labor in developing countries. As a result, stakeholders are becoming more aware of the effect of the textile, apparel, and fashion industries on the climate and human rights, thus pushing businesses to mitigate their environmental damage. This paper offers a systematic literature review of sustainability trends in the TAF industries in the last 20 years. Bibliometric tools are also used to support the content analysis of the papers. The findings reveal three primary research areas in the TAF context: consumers’ behaviour towards sustainable clothing, circular economy initiatives, and sustainability challenges across the whole supply chain. As a result, this study highlights literature gaps and provides future research suggestions for each identified research cluster. In addition, drivers and barriers to implementing corporate social responsibility and circular economy practices are identified. Consequently, this study will help researchers and academicians work in this area to identify unexplored sub-fields, which reflect some potential investigation areas for expanding scientific literature on the topic. Finally, this study supports practitioners and managers in exploring the main research themes addressed in the scientific field, providing knowledge to improve and align business models with current sustainability trends.

## Introduction

The production and consumption of clothes have consistently increased over the past few decades due to rapid population growth, increasing global incomes, and higher living standards (Shirvanimoghaddam et al., 2020). Rather than evaluating how design and production can incorporate consumer desires and sustainability, clothes are engineered and manufactured for rapid trend turnovers via obsolescence and early disposal, allowing for fast income (Kozlowski et al., 2018). This type of business model makes textile, apparel, and fashion (TAF) industries among the most polluting in the world (Grazzini et al., 2021), generating a huge volume of clothing waste (Chan et al., 2020). Indeed, less than 1% of all textiles are recycled back into clothes, 25% of textile waste is reused or recycled, and 75% of textile trash is disposed of in landfills globally (Ellen MacArthur Foundation, 2017). In terms of water consumption, the fashion industry ranks second globally (Paździor et al., 2017). In addition, the natural ecosystem suffers greatly from the dispersion into the environment of coloured effluents and microplastics, which occurs mainly in the clothing production and disposal stages (Liu et al., 2021; Sadeghi-Kiakhani et al., 2021). The COVID-19 pandemic has increased this phenomenon: the management of recently emerging wastes, often known as "COVID wastes," including cloth facemasks, is causing growing concern due to the release of microplastics into the environment (Shirvanimoghaddam et al., 2022). A potential solution to reduce the environmental consequences of cloth facemasks is using natural and biodegradable polymers for their production, such as wood-based polymers (Shirvanimoghaddam et al., 2022). In addition, textile waste can be repurposed for different applications. For instance, they can be used as a renewable source to produce thermal energy (Nunes et al., 2018). Pyrolysis is a desirable substitute for incineration in the treatment of textile waste to increase the economic benefits (Yousef et al., 2019). In addition, cotton waste can be a perfect material for creating high-performance catalysts and removing pollutants from the environment due to its natural state and affordability (Fakhrhoseini et al., 2020; Shirvanimoghaddam et al., 2019). Finally, different reusing and recycling methods for managing textile waste can be employed, such as anaerobic digestion, fermentation, composting, and fibre regeneration (Juanga-Labayen et al., 2022).

Furthermore, TAF industries account for eight to ten percentage of global greenhouse gas emissions (Shrivastava et al., 2021), recognised as the leading cause of global warming, those effects in nature create floods, droughts, hurricanes, and sea-level rise, which are becoming more common in daily life (Mishra et al., 2021). As a result, governments and institutions led to the Paris Agreement on climate change in December 2015, where 195 countries have committed to keeping the temperature rise below 2 °C (Doukas et al., 2018). Furthermore, fashion companies often entrust the transformation process of raw materials into finished clothing to developing countries, significantly affecting their social sustainability (Chan et al., 2020). Consequently, in addition to issues concerning carbon emissions, water consumption, and waste disposal, another major problem of the fashion industry is the overuse of employees working in outsourced production units in countries with unsafe work environments and lower labour costs (Shrivastava et al., 2021). According to the triple bottom line (TBL) framework, which was coined by Elkington, (1998), the performance of an apparel company should be thus measured taking into account three dimensions: economic, environmental, and social. Further, these three dimensions should be balanced, rather than just seeing economic factors as a means for society (Weisenfeld & Hauerwaas, 2018). However, in long and fragmented supply chains like apparel, harmonising these three dimensions entails difficult commitment and cooperation from different actors (Bubicz et al., 2021; Freise & Seuring, 2015; Huq et al., 2016). The longer and more complex the chain becomes, the less contact there is between the different stakeholders, and monitoring of compliance with codes of behaviour becomes more complex (Bubicz et al., 2021; Egels-Zandén et al., 2015; Macchion et al., 2015; Sardar et al., 2016; Taylor, 2011; Wilhelm et al., 2016). Government regulatory pressures are continuously coercing businesses to implement substantial changes at the technological, material, organisational, economic, and socio-cultural levels (Kivimaa et al., 2019). Further, in 2015, United Nations launched the Sustainable Development Goals (SDGs), characterised by 17 global goals and 169 targets, which aim to encourage all countries to prioritise environmental sustainability, social inclusion, and economic development (United Nations, 2015). These goals demonstrate the severity and scope of today's sustainability issues (Sauermann et al., 2020). Therefore, in order to achieve the SDGs, fashion companies must improve their corporate social responsibility (CSR) commitment in diverse areas, promoting more sustainable production and consumption models (SDG12), reducing water consumption (SDG6), and ensuring decent working conditions (SDG8).

As a result of these concerns and due to the growing interest in the United Nations SDGs, in recent years TAF industries are more prone to pay attention to sustainability issues (Islam et al., 2020; Kabir et al., 2019). TAF industries have been establishing initiatives considering eco-efficiency concepts and aspiring to implement environmental practices, including sustainability reporting activities (Muñoz-Torres et al., 2021), which generate, in turn, cost savings (Lucato et al., 2017). Improving resource efficiency by extending the useful life of products or services is one way to promote sustainable development through a more circular economy (Rainville, 2021). In this context, the product-as-a-service model, or rental model, is often associated with a number of advantages, including a decrease in the environmental impact, an improvement in competitiveness, and an increase in user value (Monticelli & Costamagna, 2022). Further, companies operating in TAF industries are looking for creative and innovative ways to keep their carbon emissions low and minimise waste (Kozlowski et al., 2018), an example is the use of biodegradable and recycled raw materials (Wang et al., 2019). Likewise, consumers are now becoming more aware of the ethical issues of the goods they purchase, and as a result, they are changing their shopping habits (De Angelis et al., 2017; Gershoff & Frels, 2015; Grazzini et al., 2021), thus pushing fashion industry to become eco-friendly.

Given the increasing attention on the topic, different researchers conducted literature reviews on sustainability in the fashion industry from diverse points of view. Notably, Koeksal et al. (2017) focused on social aspects in textile/apparel sustainable supply chain management (SSCM). Paras and Pal (2018) reviewed the literature to establish and suggest a theoretical framework for a reuse-based clothing value chain. Koszewska (2018) identified the textile sector's challenges in adapting to the circular economy (CE) model. Dordevic et al. (2019) reviewed different CSR theories and methods used in the textile/apparel industry. Wagner and Heinzel (2020) analysed the literature on CE in the fashion industry, focusing on consumer behaviours concerning the sustainable purchase, usage, consumption, and disposal. Islam et al. (2020) summarised the primary environmentally friendly practices adopted by TAF industries. Jia et al. (2020) identified drivers, barriers, strategies, and performance measures for the CE in the fashion industry. Finally, Ki et al. (2021) reviewed the literature to provide a theoretical framework that offers a detailed explanation of how fashion companies can achieve circularity by involving external stakeholders in their activities.

Based on the above premises, in the scientific literature, there is a lack of literature reviews that offers a holistic understanding of sustainability issues in the TAF industries and evaluates research advances and trends on the topic to benefit multiple stakeholders. This paper aims to overcome these research gaps with a comprehensive overview of sustainability trends in the TAF manufacturing context. In addition, this research highlights both CSR and CE principles, supporting academicians, policymakers, practitioners, and other decision-makers in exploring the main research themes addressed in the scientific field. This paper is expected to contribute to the literature in the following ways. First, this study addresses the research gaps by offering a holistic perspective of a study area that is rapidly expanding. Second, this research combines the review process with bibliometric techniques. Although the growing interest in the research field, these approaches have not yet been adopted to explore sustainability progress in the TAF industries. Third, drivers and barriers to implementing CSR and CE practices are identified. Notably, CE is a production and consumption model that aims to extend products’ useful lives by helping to minimise waste, while CSR is often described as corporate practices that address economic, social, and environmental issues to benefit citizens, communities, and societies. The proposed taxonomy could be a reference point for further empirical studies. Finally, this article develops a conceptual model based on the extracted research clusters that integrate previous research findings, highlight research gaps, and offers guidance and potential avenues for further research to fill in the literature gaps.

After this introduction, Sect. 2 describes the review methodology adopted. Section 3 shows the data collection and selection phase. Sections 4 and Sect. 5 highlight descriptive and content analysis of the articles. Section 6 reports research discussions and provides a detailed research agenda. Finally, Sect. 7 presents conclusions and implications, highlighting theoretical and managerial contributions, as well as the research policy implication.

## Review methodology

This study presents a systematic literature review adapted by Greenhalgh (1997), Cerchione and Esposito (2016), and Centobelli et al. (2017). Therefore, according to these contributions, we structured the literature review into two primary phases:

1. Data collection and selection: this phase includes identifying keywords and the search string, choosing the academic database (e.g. Scopus and Web of Science) to retrieve documents, and defining the inclusion/exclusion criteria to obtain papers focused on the research topic examined
2. Descriptive and content analysis phase: this phase includes conducting descriptive statistics (e.g. papers over time and articles by methodology) and an in-depth content analysis of the selected papers, aiming at identifying research gaps and providing a research agenda for further investigation.

**Fig. 1:**
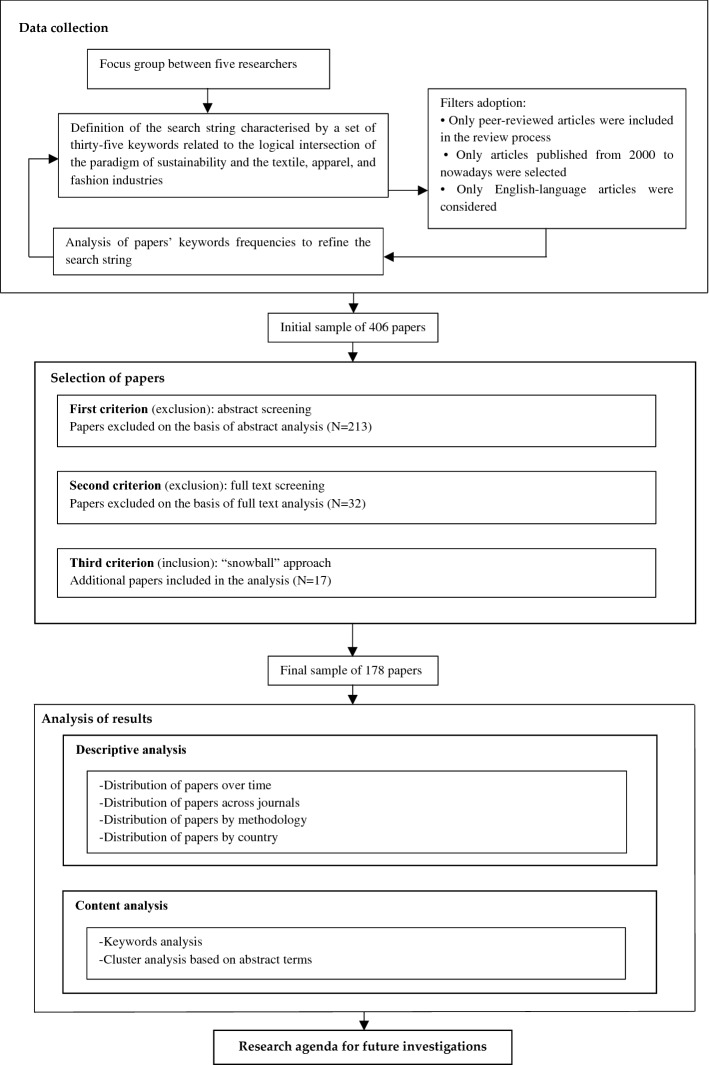
Literature review methodology

Furthermore, we applied bibliometric methods to support the content analysis phase (van der Have & Rubalcaba, 2016). Notably, bibliometric techniques represent powerful tools to analyse scientific literature in a specific research field quantitatively (Ji et al., 2018; Zhi & Ji, 2012). One of the primary bibliometric methods is science mapping (Dzikowski, 2018) and it was used to discover the research field structure of a given topic (Cancino et al., 2017; Merigó et al., 2017; Shashi et al., 2021). This analysis can be implemented through numerous computer software. In this paper, we used VOSviewer software to build and visualise co-occurrence networks of keywords and paper terms, showing the main topics studied and suggestions for future research (Liboni et al., 2019). In particular, the co-occurrence analysis of keywords is an effective method for identifying research themes since it helps analyse the paper's content and assess the co-occurrence relationship between different concepts (Shashi et al., 2020a, 2020b). Furthermore, the co-occurrence network of abstract terms is used to show research clusters based on recurrent terms that appear together (Liboni et al., 2019). According to van der Have and Rubalcaba (2016), the higher the frequency that keywords and paper terms co-occur, the stronger they are linked because they belong to a similar research sub-area. Thus, we aim to overcome this lack by offering a comprehensive literature review. Figure 1 synthesises the steps of the proposed literature review methodology.

## Data collection and selection

The sample of articles was retrieved from the ISI Web of Science (WoS) database. More specifically, the WoS Core Collection was used in this study. Due to the high quality and extensive background coverage, the WoS database has traditionally been used as the primary source for literature reviews (Alon et al., 2018; Bahoo et al., 2020; Cao & Alon, 2020). Moreover, WoS is considered a leading data source compared to other scholarly research databases (e.g. Scopus and Google Scholar) since it only contains selective journals (Shashi et al., 2020b). More precisely, WoS includes over 15,000 high-quality journals and 50,000,000 papers, organised into 251 categories and 150 research topics (Gaviria-Marin et al., 2019; Shashi et al., 2020a).

After a brainstorming process among five researchers, a list of keywords was identified to carry out a systematic search and find articles regarding the issue of sustainability in the TAF industries. Further, the list of keywords was refined from time to time by including the keywords of the papers found previously. Finally, the following search string was used:

("textile industr*" OR "textile sector*" OR “clothing” OR “clothes” OR “garment” OR “fashion” OR “apparel”) AND (“green” OR "environmental performance" OR "financial performance" OR "social performance" OR “green” OR "economic* performance" OR "environmental benefit*" OR "financial benefit*" OR "economic* benefit*" OR "social benefit*" OR "ethical" OR "SDG*" OR "sustainable development" OR "corporate social responsibility" OR "triple bottom line" OR "environment-friendly" OR "eco-friendly" OR "circular economy" OR “reuse” OR "re-use" OR “recycling” OR "life cycle assessment" OR "life cycle analysis" OR “LCA” OR (“sustainab*”)) AND (“environment*” OR “economic*” OR “social”). We retrieved only documents that contain those terms in the title to circumscribe the research and identify only relevant outputs on the topic investigated.

To perform bibliometric analyses, we downloaded the full record and cited references of scholarly articles in the Web of Science Core Collection (Kern et al., 2019). The sample of 563 documents was retrieved in October 2022. We used different filters to refine our analysis. First, we chose to not consider papers published before 2000 due to the actuality of the topic (Desore & Narula, 2018) and we aim to conduct a review of the last two decades. Second, we collected only papers written in the English language (Shashi et al., 2020a, 2020b). Subsequently, to ensure the sources’ quality, we decided to select only articles and reviews published in peer-reviewed journals, thus excluding other types of sources such as conference proceedings and book series (Shashi et al., 2020b). As a result, 406 papers were collected.

Furthermore, according to the method suggested by Pittaway et al. (2004), we carefully checked the abstracts of all the selected papers so that only those studies whose abstracts focus on sustainability in the TAF industries were selected. To avoid subjective decisions, two researchers read the abstracts of the articles in parallel, with the intervention of a third researcher in case of uncertainty (Cerchione & Esposito, 2016). Thus, as also displayed in Table 1, the papers were divided into the following two lists: list A includes documents whose abstract focuses on sustainability in the TAF industries and list B includes documents whose abstract focuses on technical and context-specific aspects of sustainability (e.g. processing, atmospheric emissions due to production waste, the chemistry of eco-sustainable fabrics)

**Table 1:** Articles’ selection based on abstract

List	Description	Number of papers
A	Documents that focus on the textile, apparel, and fashion industries' sustainability	193
B	Documents that focus on technical and specific aspects of sustainability	213
Total		406

The articles contained in list B (213) were excluded as beyond the scope of the research. The full text of the 193 articles included in list A were thoroughly examined and subjected to the last exclusion criterion. Also, in this case, two researchers read the papers in parallel, plus a third one in case of doubt (Cerchione & Esposito, 2016). In this step, we excluded 32 documents not related to the research topic. To identify the remaining potentially important studies in our set, we used the 'snowball' strategy as an inclusion criterion (Greenhalgh & Peacock, 2005). We included 17 additional publications, and the final sample thus consists of 178 papers.

## Descriptive analysis

The purpose of the descriptive analysis is to provide a general view of the papers on sustainability in the TAF industries. For the evaluation of the 178 papers selected, four viewpoints were identified: 1) distribution of papers over time; 2) distribution of papers across journals; 3) distribution of papers by methodology; and 4) distribution of papers by country.

### Distribution of papers over time

**Fig. 2:**
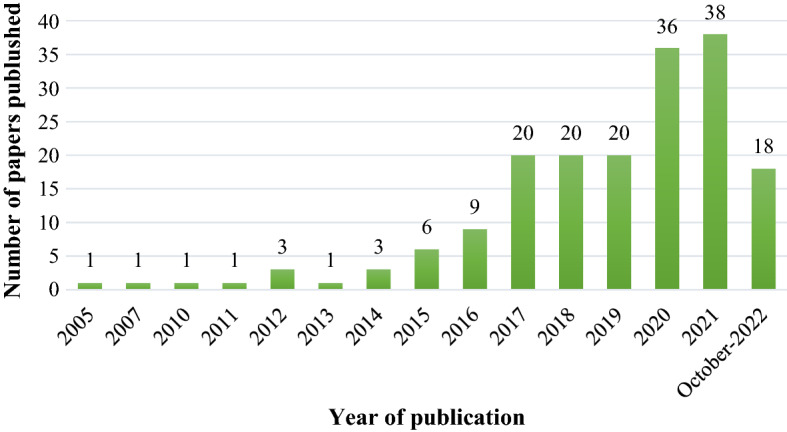
Papers over time

Figure 2 shows the distribution of the selected papers published between 2000 and 2022. The number of papers written has grown exponentially, reaching a maximum of 38 in 2021. The data collection was conducted in October 2022. According to this analysis, in the last five years, research on sustainability in TAF industries has grown significantly. Indeed, approximately 85% of the papers examined were written between 2017 and October-2022.

### Distribution of papers across journals

**Fig. 3:**
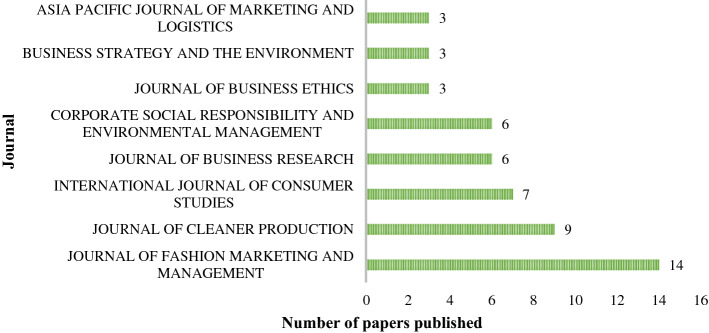
Papers published per journal

The journals that published at least three papers on sustainability in the TAF industries from 2000 to October 2022 are classified in Fig. 3. The top journals publishing on the research topic have a broader scope and belong to different areas, confirming that the analysis of sustainability issues in the TAF industries have grown over the years in a broader range. In particular, *Journal of Fashion Marketing and Management* (14), followed by *Journal of Cleaner Production* (9), *International Journal of Consumer Studies* (7), *Journal of Business Research* (6), and *Corporate Social Responsibility and Environmental Management* (6), *Journal of Business Ethics* (3), *Business Strategy and the Environment* (3), and *Asia Pacific Journal of Marketing and Logistics* (3). According to the SCImago Journal Rank (SJR) updated to the year 2020, used to determine each journal's scientific importance, all of the journals displayed in Fig. 3 are in the first quartile (Q1), except for *Asia Pacific Journal of Marketing and Logistics* and *International Journal of Consumer Studies*, which are in the second quartile (Q2).

### Distribution of papers by methodology

**Fig. 4:**
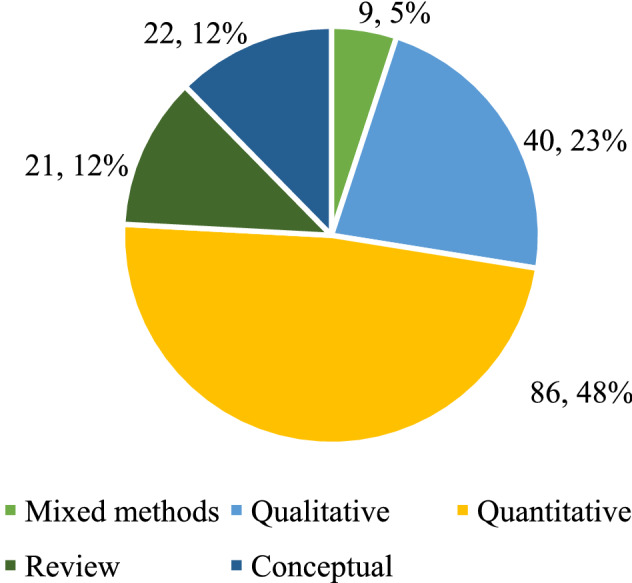
Papers by methodology

The distribution of papers by methodology represented in Fig. 4 shows that about 50% of the studies are based on quantitative approaches (e.g. surveys and mathematical models), while 23% of the papers use qualitative approaches (e.g. single and multiple case studies). A few other papers use conceptual approaches, literature review approaches, and mixed approaches (combining qualitative and quantitative methods).

### Distribution of papers by country

**Fig. 5:**
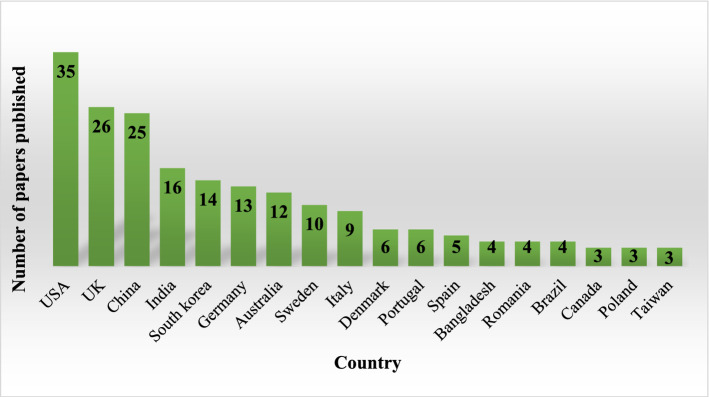
Papers by country

This analysis highlights the most productive countries in the research field investigated. Notably, certain papers were co-authored by researchers from different countries, while authors from the same nationality co-authored others. The country of each researcher who co-authored the article is counted in the first situation. On the contrary, the country is only counted once, even if two or more researchers from the same country co-authored the paper. As shown in Fig. 5, USA is at the top of the ranking with 35 publications, followed by the UK (26), and China (25).

## Content analysis

### Keywords analysis

**Fig. 6:**
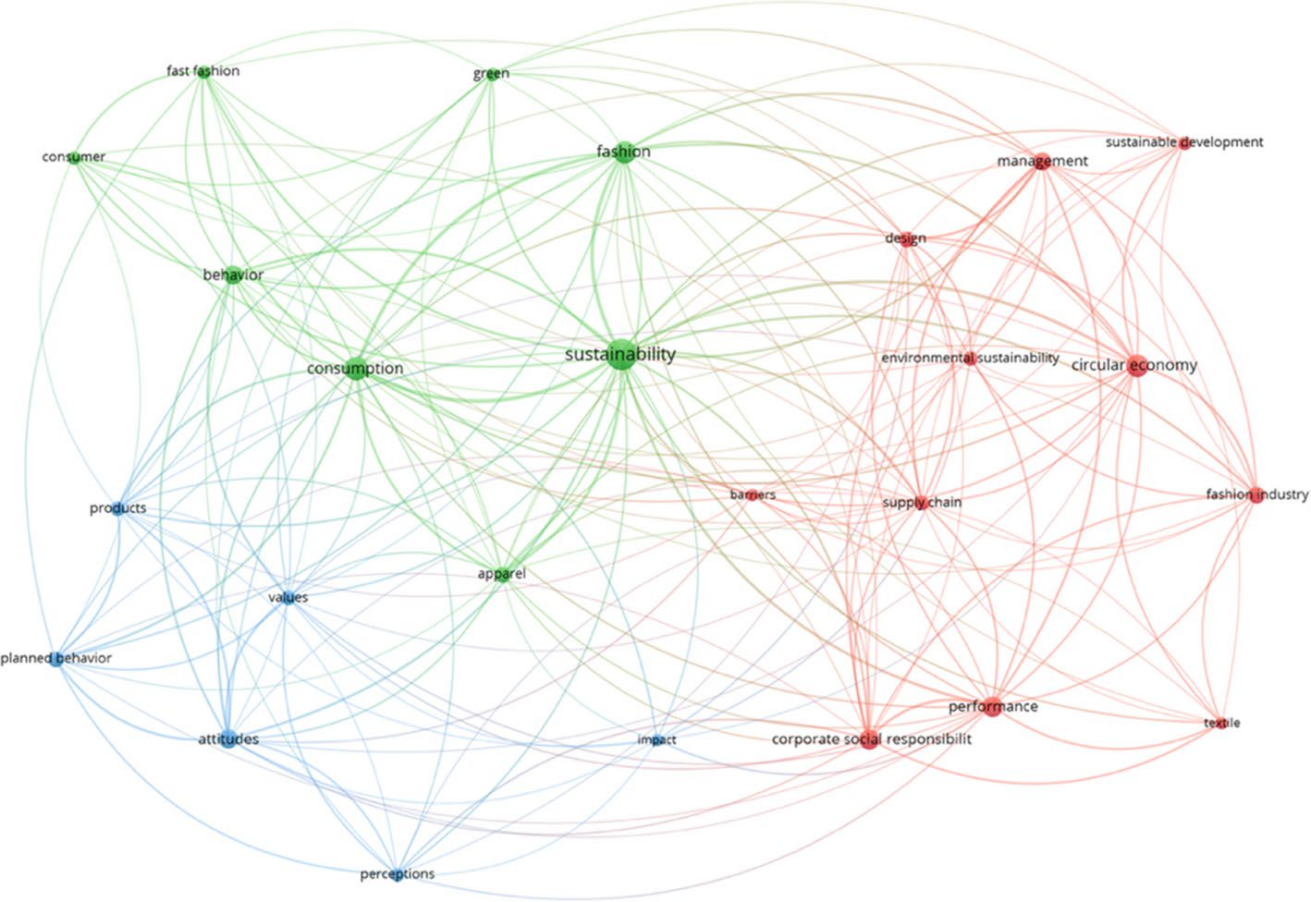
Co-occurrence analysis of keywords

This analysis found 833 different keywords in the sample of 178 papers. The research focused on keywords that had at least eight repetitions (Liboni et al., 2019). Consequently, a total of 25 unique keywords were chosen (Fig. 6). In particular, the keyword “sustainability” emerged as the most recurrent with 45 repetitions, followed by “consumption” (26), “circular economy” (24), “fashion” (23), and “corporate social responsibility” (20).

“Appendix 1" highlights the 20 most cited keywords and their total link strength. The most frequent keywords offer an in-depth understanding of the critical topics investigated. Furthermore, the keyword sustainability is strongly linked with the others, and its relationship with "fast fashion", "supply chain", and "consumption" highlights that scientific literature in the TAF domain is extensively focused on studying more sustainable business models which can reduce the environmental footprint in all the phases of the supply chain. The term “fast fashion” refers to a business model defined by constant shift, innovation, affordability, and disposable patterns concerning low-cost apparel products that replicate existing luxury fashion trends (Joy et al., 2012). Diverse scientific studies have confirmed that fast fashion's disposal nature leads to serious environmental, health, social, and economic issues (Shirvanimoghaddam et al., 2020). As a result, various alternative business models have been developed. For instance, the clothing product-service system (PSS) recognises various sustainability targets as an alternative to the effects of consumption and fast fashion (Johnson & Plepys, 2021). This business model is based on rental rather than purchase, allowing to extend the useful life of a garment and reduce waste. Thus, PSS shifts the emphasis to complementary service offerings, which dematerialises and decouples consumer loyalty from material use (Adam et al., 2017). Another primary problem for fashion companies' is the supply chain length and complexity, causing coordination and sustainability concerns. According to Carlson and Bitsch (2018), a sustainable supply chain is a crucial element for industry, government, and civil society. Recent research highlights that fashion retailers often engage procurement intermediaries to handle their international sourcing with suppliers from manufacturers operating in developing countries, improving coordination and transparency (Koeksal et al., 2018).

### Cluster analysis based on abstract terms

**Fig. 7:**
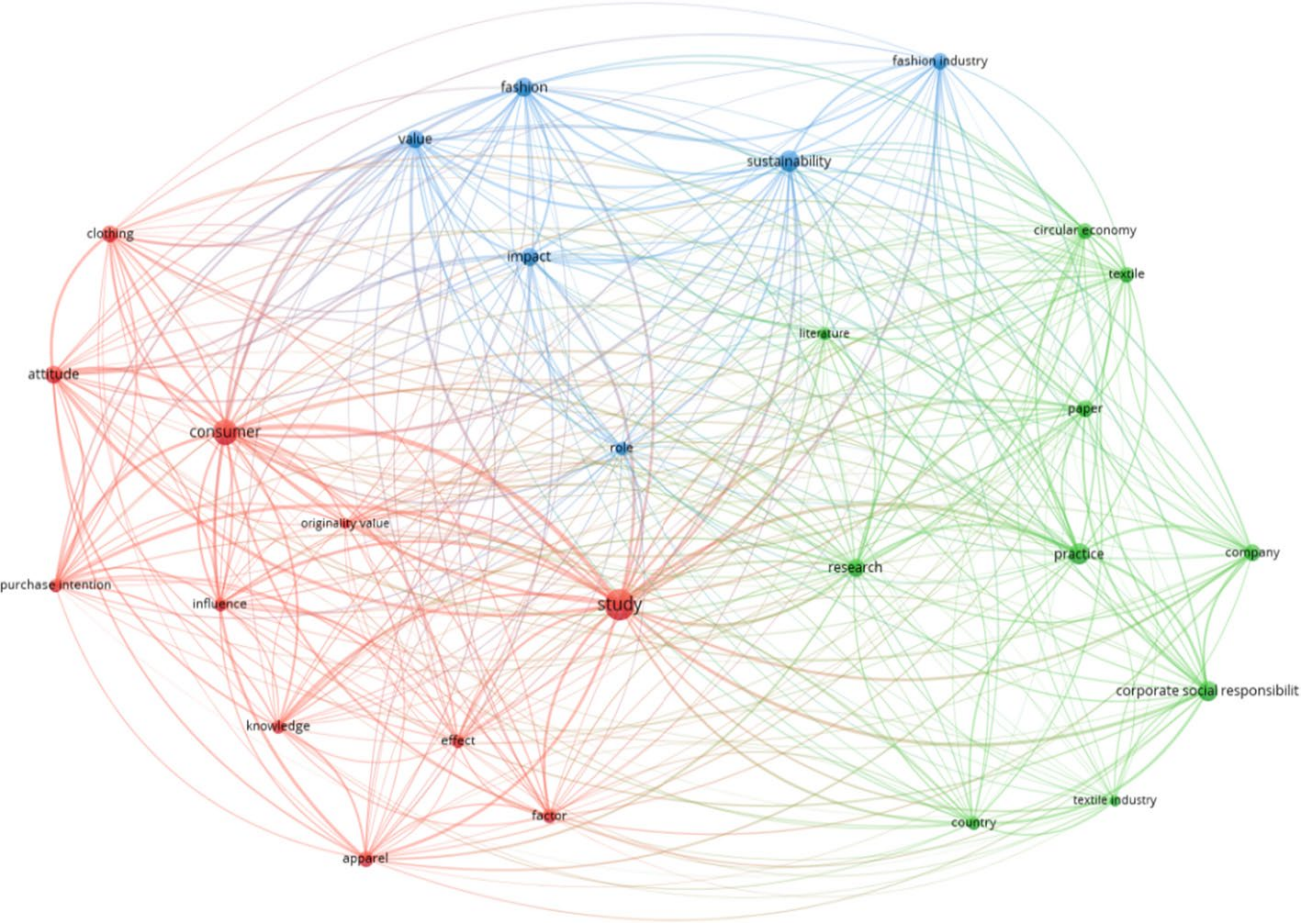
Co-occurrence network of abstract terms

In our sample of articles, the co-occurrence analysis of abstract terms has shown 3657 different recurrent words. However, we selected only terms with at least nine repetitions and just 27 terms resulted in the analysis (Liboni et al., 2019). Figure 7 displays its network visualisation. “Appendix 2” highlights the 20 most recurrent abstract terms and their relevance score. Using VOSviewer, we divided the abstract terms into three different research clusters:

- Cluster 1: consumer behaviour concerning sustainable clothing consumption
- Cluster 2: circular economy and corporate social responsibility issues in the TAF industries
- Cluster 3: impact of sustainability initiatives on corporate performance.

#### Consumer behaviour concerning sustainable clothing consumption

This cluster is focused on sustainable clothing consumption through the lens of consumer behaviour. The consumer's vision of eco-sustainable clothing is a central theme in the literature on TAF industries. The previously reserved attention for an elite audience is now directed to an increasingly widespread profile of evolved consumers who are more interested in the origin of what they buy and the traceability of the supply chain. Therefore, the customer plays a crucial role in the sustainable context, so it is vital to understand his point of view towards eco-sustainable, recycled, or used products. The majority of contributions included in this cluster are surveys, in which the relationship between the constructs was tested chiefly through structural equation modelling (SEM). In light of the theory of planned behaviour, most of these studies investigate the factors influencing sustainable apparel purchase intention (e.g. Dhir et al., 2021; Hwang et al., 2020; Kang et al., 2013; Karaosman et al., 2015; Nguyen et al., 2019; Sobuj et al., 2021; Zhao et al., 2019), revealing that sustainable clothing buying is positively correlated with different antecedents, such as green confidence, environmental awareness, social media usage, environmental attitude, labelling satisfaction, subjective norms, and perceived behavioural regulation. Other studies focused specifically on the young generations. For example, Varshneya et al. (2017) surveyed young consumers to explore how green consumption principles and social influence affect buying organic clothing. In particular, using a multinominal logit model, Rothenberg and Matthews (2017) determined the primary factors influencing young consumers to buy eco-friendly t-shirts. The findings suggest that consumers prioritised price, followed by the location of production, and finally, sustainability issues. Other studies analysed the existing attitude-behaviour gap and examined consumers' barriers to buying green clothing (Jacobs et al., 2018; Wiederhold & Martinez, 2018). Finally, Byrd and Su (2020) surveyed 399 US consumers to discover how they feel about apparel brands and how they behave when it comes to environmentally friendly, affordable, and socially conscious clothing. Further research focused on the motivations for using sharing economy platforms (Lee & Huang, 2020; Ek Styvén and Mariani, 2020), indicating that consumers' intentions to use online fashion rental services were positively affected by different factors, such as behaviours, subjective norms, perceived environmental sustainability, economic motivation, and distance from the consumption system. On another note, Silva et al. (2021) revealed that social shame and consumers' lack of knowledge about available outlets are the factors that most negatively affect the purchasing of second-hand clothing.

Moreover, many studies examined recycled and reused products from various perspectives. Some researchers investigated how consumers handle their apparel waste, including reselling, swapping, taking back, and donating (Lai & Chang, 2020; Weber et al., 2020), highlighting that environmental principles and prosocial attitudes affected customers' decisions to donate clothes. Other studies surveyed random samples to investigate consumer recycling and reusing apparel behaviour (Paco et al., 2021; Zurga et al., 2015). Further, Park and Lin (2020) examined the discrepancy between purchasing purpose and purchase experience in recycled and upcycled fashion items. Other studies focused on behaviour intentions for the consumption of reused clothing, recycled clothes, and upcycled garments (Chaturvedi et al., 2020; Kim et al., 2021). Notably, Meng and Leary (2021) explored consumer perception concerning the transformation of recycled bottles into new clothes. Consumers perceive this practice negatively for hygienic reasons, reducing purchasing intent. Finally, Cruz-Cardenas et al. (2019) conducted a multiple case study based on 20 thorough interviews followed by a survey of 425 consumers to investigate the factors affecting clothing reuse, highlighting different antecedents, such as income and altruism.

#### Circular economy and corporate social responsibility issues in the TAF industries

This cluster concerns CE and CSR issues in the TAF industries. Unlike the previous one, this cluster includes many explorative studies since the CE and CSR implementation is still in its early stages, necessitating a more detailed understanding based on qualitative analysis (Colucci & Vecchi, 2021). In particular, the CE principles significantly improve sustainability in the way textile products are fabricated, consumed and disposed of (Staicu & Pop, 2018). Different frameworks have been developed to help fashion companies transition from a linear to a CE model (e.g. Mishra et al., 2021). Indeed, several critical factors need to be explored for developing a circular product in the textile industry context, such as sustainable product design and reverse logistics (Franco, 2017). Concepts such as repairability, recyclability, longevity, and reuse and disposal of products are much debated in the literature. Although they are still at the early stages, different methods for reusing, recycling, and regenerating textile waste as well as various technological innovations and plans for a circular textile economy have been developed (Shirvanimoghaddam et al., 2020). In this regard, Moazzem et al. (2021) used the life cycle assessment (LCA) methodology to evaluate environmental benefits due to different textile waste recycling opportunities. The findings show that cleaning wipes recycling has the most significant impact benefits, followed by cotton fibre, insulation material, and polyester raw material recycling. Sandvik and Stubbs (2019) conducted a multiple case study based on semi-structured interviews to determine drivers and barriers to implementing a textile-to-textile recycling technology in the Scandinavian fashion industry. Restricted technology (which makes separating materials difficult), high research and development costs, and the supply chain complexity (which includes many stakeholders involved in the manufacture), represent the key barriers. Simultaneously, the design and use of new fabrics and increased apparel collection and collaboration are the main drivers.

Furthermore, many studies used a case study approach to investigate the challenges and solutions that fashion brands face while developing and testing CE strategies within their current business models (Kant Hvass & Pedersen, 2019; Colucci & Vecchi, 2021). The findings show that fashion companies face several obstacles in implementing circular business models in their organisations, including divergent perspectives of value and undefined performance metrics, weak alignment with the current strategy, a lack of internal skills and competencies, and a lack of customer interest. Further, Paras et al. (2018) conducted a multiple case study based on semi-structured interviews with Swedish companies to explore the reuse-based clothing value chain drivers. The results suggest that the main drivers are corporate factors (system, legislation, and awareness), product features (design, quality and price), and consumer attitude (donor and purchaser).

Other studies focused on the slow fashion movement. According to Onur (2020), the slow fashion movement believes that the fashion industry should not continue operating in the same way it has in the past, putting the world’s finite resources at risk. As a result, the author offered a detailed account of creating new learning methods and designing via upcycling, craft, and collaboration in developing countries. For instance, Friedrich (2021) investigated the potential of applying biobased products in the textile industry, making the economy more sustainable and lowering the dependence on synthetic materials. Tama et al. (2017) surveyed Turkish university students to investigate clothing awareness and attitudes regarding environmental sustainability and slow fashion, and the findings highlighted a lack of knowledge about the slow fashion paradigm.

Moreover, some of the studies analysed circular business models based on clothing swapping, PSSs, and collaborative fashion consumption (CFC). Notably, clothing swapping is an example of a circular solution that allows extending the useful life of a product (Camacho-Otero et al., 2020), while the CFC is an economic model focused on clothing sharing, second-hand purchases, and renting or leasing (Zamani et al., 2018). Compared to a traditional ownership-based consumption model, the CFC offers environmental benefits due to the extension of the clothes’ useful life. On another note, Bech et al. (2019) used the LCA approach to assess and compare a PSS business model’s environmental impact on t-shirts and a reference business model.

Furthermore, different studies used the multiple case study design to examine CSR strategies’ drivers and barriers (Govindasamy & Suresh, 2018; Guedes et al., 2017; Koeksal & Straehle, 2021; Van & Nguyen, 2019), showing that the main drivers are the competitive context, the social influences, the managers’ knowledge of CSR, the company’s internal culture, as well as market promotion and building a reputation with stakeholders and the government. Additionally, the most significant obstacles were a lack of resources in expertise, information, finance, and training, as well as the cost of CSR initiatives and internal and external communication. Further obstacles were the complexity of the green process and system design, as well as the lack of regulatory support (Majumdar & Sinha, 2018).

#### Impact of sustainability initiatives on corporate performance

The studies of this cluster investigated how different sustainable initiatives affect corporate performance (Chan et al., 2020; Saha et al., 2021; Sudusinghe & Seuring, 2020; Wong & Ngai, 2021; Yang & Jang, 2020). In particular, Ali et al. (2020) revealed that fashion companies that successfully implemented ISO 14001 environmental management system (EMS) reported substantial efficiency improvements compared to companies that have not yet EMS.

Specifically, some studies focused on the sustainable supply chain, which is achieved when the objectives are shared by all the actors involved. This entails reconsidering production flows, operations, and materials, limiting the polluting effects that flow into the environment, limiting production waste, extending the life cycle of the products, and improving social conditions. Kumar et al. (2020) used the Delphi-based fuzzy Analytical Hierarchy Process approach to identify long-term factors for implementing social responsibility-based sourcing in the ready-made apparel supply chain in Bangladesh. Further, Ashby (2018) used an in-depth case study to explore how a closed-loop supply chain (CLSC) can improve the environmental performance of a UK clothing company. The results highlight the crucial role of strategic resources and a shared vision and culture among the company and its suppliers, from a more reactive environmental damage prevention plan to a comprehensive CLSC. Jesus Munoz-Torres et al. (2021) used the LCA method to quantify textile companies’ environmental impact throughout the supply chain and compare their performance with global and sectorial sustainability challenges. The findings reveal a connection between global environmental issues and corporate environmental disclosure.

#### Taxonomy of CE and CSR drivers and barriers

Based on the previous literature, Table 2 highlights the main factors which potentially affect the propensity of fashion companies to adopt CE and CSR principles, as well as the main barriers hindering their implementation. The proposed taxonomy might serve as a starting point for more empirical research.

**Table 2:** CE and CSR drivers and barriers

	Drivers	Barriers
Corporate social responsibility	Competitive context Social influences Knowledge of CSR Internal culture Business promotion Reputation with stakeholders and government Customer environmental awareness	Cost of CSR initiatives Difficulties in internal and external communication Difficulties in implementation Lack of resources in expertise, information, finance, and training Complexity of CSR Supply chain lacking transparency
Circular economy	Development of new types of fabrics Clothing unique design Increased apparel collection Corporate awareness Legislation Consumers’ environmental consciousness Customer distance from consumption models	Divergent perspectives of value Undefined performance metrics Weak alignment with the current strategy Lack of internal skills and competences Lack of customer interest Restricted technology High research and development costs Supply chain complexity

## Discussions and future research directions

The descriptive analysis provided a general overview of the articles included in the literature review, highlighting that, in recent years, there is growing attention on sustainability in the TAF industries and that these topics present different scopes, belong to different disciplines, and are covered by different journals.

The content analysis of the selected articles highlighted the literature’s strengths and weaknesses, thus identifying current research and providing research ideas for future investigation. It is possible to classify the selected papers into five main research areas: 1) consumer behaviour; 2) circular economy; 3) corporate social responsibility; 4) business models; and 5) supply chain management. Table 3 offers a more in-depth discussion of existing research and future research suggestions for each of these scientific areas.

**Table 3:** Current research and future research suggestions

Research area	Current research	Future research suggestions
Consumer behaviour	Factors influencing sustainable clothing purchasing (e.g. Nguyen et al., 2019; Hwang et al., 2020; Dhir et al., 2021; Sobuj et al., 2021; Sobuj et al., 2021) Factors influencing clothing reuse and clothing disposal behaviour (e.g. Cruz-Cardenas et al., 2019; Paco et al., 2021; Park & Lin, 2020; Zurga et al., 2015)	Eco-design characteristics influencing ethical clothing consumption Effectiveness of social network communication tools adopted by TAF firms to encourage sustainable clothing consumption Meta-analysis to summarise the results from primary quantitative studies on sustainable consumer behaviour
Circular economy	Drivers and barriers to implementing circular economy strategies in the TAF industries (e.g. Kant Colucci & Vecchi, 2021; Hvass & Pedersen, 2019) Implementation of the LCA methodology to quantify companies’ environmental impact throughout the supply chain (e.g. Bech et al., 2019; Jesus Munoz-Torres et al., 2021; Moazzem et al., 2021)	Development of new technologies for sorting textile waste Measuring the environmental and economic impact of different circular/sustainable products using the LCA-LCC methodology
Corporate social responsibility	Drivers and barriers to implementing corporate social responsibility practices in the TAF industries (e.g. Govindasamy & Suresh, 2018; Guedes et al., 2017; Koeksal & Straehle, 2021; Van & Nguyen, 2019)	Analysis of stakeholder concerns for the implementation of a holistic sustainability strategy
Business models	Clothing product-service system (e.g. Adam et al., 2017; Johnson & Plepys, 2021) Fast fashion business model (e.g. Shirvanimoghaddam et al., 2020)	Analysis of the shift of paradigm from fast fashion to slow fashion
		Analysis of circular innovations upstream and downstream of the TAF supply chain
Supply chain management	Sustainability challenges along the TAF supply chain (e.g. Ashby, 2018; Kumar et al., 2020)	Digital technologies’ role in improving supply chain transparency

The first research area discusses the drivers influencing sustainable apparel purchasing (e.g. labelling satisfaction and environmental awareness), clothing reuse (e.g. income and altruism), as well as different clothing disposal behaviour (e.g. donation and recycling). Firstly, future research could perform a meta-analysis to generalise the empirical results of previous quantitative investigations on sustainable clothing consumer behaviour, thereby obtaining more robust conclusions than those drawn from each study. Further, as the production activities, business processes and materials contribute to an increase in the global pollution rate, eco-design features, ecological materials, processes with low environmental impact, and waste reduction have been developed in recent years (Heinze, 2020). This area shows the need for a more in-depth analysis of the eco-design characteristics that positively influence the ethical clothing consumer’s purchase intentions. Further, there is also a lack of studies investigating the efficiency and effectiveness of the communication tools adopted by TAF companies to encourage consumers to purchase sustainable clothing. For instance, compared to traditional channels, such as reports and advertising campaigns, corporate websites are constantly being used to present the business’ formalised and official viewpoint on CSR activities (Mann et al., 2014). The consumers’ opinion on this aspect could therefore be more in-depth analysed in further investigation.

The second research area focuses on drivers and barriers to adopting CE strategies in the TAF industries. In the TAF industries, due to the variety of fabrics and clothing accessories used, such as buttons and zips, end-of-life textiles are difficult to handle after disposal (Marques et al., 2020). Since there are presently few technologies available for separating recyclable textile waste from non-recyclable textile waste, employees still do much of the job by hand (Centobelli et al., 2022). Future studies could therefore design and develop new technological advances for managing and sorting textile waste. Automating the process and launching it on an industrial scale will therefore be the key to a real revolution in the world of fabrics.

Furthermore, many of the articles we analysed use the LCA methodology to evaluate companies’ environmental impact throughout the supply chain. However, there is a lack of studies examining the environmental and economic impact of different sustainable and circular clothing using the LCA and Life Cycle Costing (LCC) methodologies. Indeed, the integration of these methods will provide a holistic understanding of sustainable clothing production, allowing companies to choose materials that guarantee greater added value and which at the same time respect the environment.

The third research area is mainly focused on CSR drivers and barriers. Organisations require greater attention to social and environmental issues to develop a successful business. As a result, companies are changing their modus operandi, developing sustainable initiatives from a social and environmental point of view. According to Zhu et al., (2016), businesses are under pressure from stakeholders to reduce the negative environmental impact they generate while increasing CSR initiatives. Companies recognise the strategic importance of reacting to stakeholder concerns as a means of strengthening their competitive position (Zhu et al., 2016). Consequently, future studies on the analysis of stakeholder concerns in the context of TAF industries are needed to develop a holistic corporate sustainability strategy.

The fourth research area discusses different types of business models in the field of TAF industries. A vast majority discusses the PSSs and the fast fashion model. However, this area highlights the need for a more comprehensive analysis of the slow fashion business model. Slow fashion is based on various principles, such as the quality of the products, the recycled and eco-compatible materials, and the short supply chain (Jung & Jin, 2016). Consequently, this type of business requires greater awareness of consumers and manufacturers, as it tends to reduce the production cycle and consequently consumption. Slow fashion is aimed at safeguarding the climate, workers, natural resources, and the economy. However, due to the higher costs of slow fashion products compared to mass-produced clothes, the potential of slow fashion to make and maintain a profit represents a critical point that should be explored better. It is necessary to investigate the external pressures affecting the development of the slow fashion business model, also considering all the issues related to the transition to this new type of business model. Further, there is a lack of studies examining the circular business model innovations in the TAF industries (Henry et al., 2020). More in detail, according to the taxonomy proposed by Urbinati et al., (2017), three types of circular companies can be identified: downstream, upstream, and full circular companies. Downstream circular businesses follow a pricing scheme or a marketing strategy focused on product use and re-use, but these contributions neglect the necessary changes at the supplier level or internal processes or product design. On another note, upstream circular companies are described as those that implement circular solutions internally (e.g. using recycled raw materials) and focus on the interactions with their suppliers. Finally, full circular companies implement both downstream and upstream circular business model innovations. As a result, future studies could examine the degree of circularity of the TAF companies, analysing if circular business model innovations are implemented downstream, upstream, or both.

Finally, the fifth research area mainly focuses on the analysis of different social and environmental sustainability challenges along the fashion supply chain. From this research area emerged the need to explore the role of digital technologies in improving sustainability performance. Indeed, digital enabling technologies like blockchain can guarantee the complete traceability and transparency of products, thus optimising the entire supply chain and improving company performance (Centobelli et al., 2021). Consequently, these technologies could be an excellent resource for TAF companies, representing a strategic tool for environmental protection and sustainable development and facilitating the spread of sustainable practices.

## Conclusions and implications

### Contribution to the theory

This paper offers a comprehensive analysis of sustainability trends in the TAF industries, providing different theoretical contributions and extending the results provided by previous research. We adopted bibliometric techniques (i.e. co-occurrence analysis of keywords and abstract terms) to support the content analysis phase of the review methodology and provide quantitative insights offering a holistic understanding of the research field, integrating CSR and CE aspects. Notably, the co-occurrence network of abstract terms revealed three main research clusters: (1) consumer behaviour concerning sustainable clothing consumption, (2) circular economy and corporate social responsibility issues in the TAF industries, and (3) sustainability challenges in the fashion industry. By thoroughly analysing these clusters, we developed a conceptual framework which integrates prior study findings, identifies research gaps, and provides potential directions for future research. Consequently, this study will help researchers and academicians work in this research area to identify unexplored sub-fields, which reflect some potential investigation areas for expanding scientific literature on the topic. Moreover, the proposed taxonomy of CE and CSR drivers and barriers in the fashion industry context could be used by researchers in future investigations as a reference point for conducting empirical studies.

### Contribution to practice

This study offers different opportunities to the public authorities, businesses, and practitioners involved in the path towards sustainability in the TAF context. It provides a broad range of relevant knowledge regarding how sustainability and circularity principles are affecting TAF industries. Such knowledge is essential for managers of TAF industries since it allows them to innovate their business models and prosper in today's competitive environment, thus moving to less polluting production systems and improving company performance. Manufacturing companies, purchasing organisations, and other stakeholders could gain a deeper understanding of the problems, procedures, predictors, barriers, and challenges associated with implementing sustainable practices and developing the skills necessary to reduce environmental impacts and gain competitive advantages.

Furthermore, this study may have political implications. It is acknowledged that the TAF industries represent a major source of environmental pollution. Therefore, the results of this study may inspire governments to promote sustainable initiatives in the TAF industries. For instance, policies implemented by the governments may include incentives for using eco-sustainable and recycled materials or financing for the purchase of green technologies with a lower environmental impact. In addition, for TAF industries to achieve the SDGs, the government must promote cultural changes that move innovation from an individualistic logic bound only to profit maximisation to a collectivistic, communal and open logic based on sustainable development principles.

### Limitations of the study

Although considerable attention was taken to ensure the study process's validity and outcomes, certain limitations must be acknowledged. First, despite we adopted a validation criterion to integrate papers published in different academic databases, we limited our initial search to papers published in the WoS database. Furthermore, we just looked at papers and reviews published in peer-reviewed journals, ignoring other types of publications, including conference proceedings and book chapters. Second, we used VOSviewer software to conduct the co-occurrence analysis of keywords and paper terms, but other statistical analysis and clustering methods can be used, such as coauthorship analysis. Another limitation is regarding the related concept (i.e. zero waste), which is not incorporated within the scope of this research. Further studies can expand the scope to such related concepts/theories.

## Data Availability

The datasets generated during and/or analysed during the current study are available from the corresponding author upon reasonable request.

## Appendix 1. Frequently used keywords

**Table:**

Keywords	Frequency	Total link strength	Keywords	Frequency	Total link strength
Sustainability	45	131	Design	12	42
Consumption	26	87	Planned behaviour	12	42
Circular economy	24	56	Products	11	35
Fashion	23	70	Environmental sustainability	10	35
Corporate social responsibility	20	44	Supply chain	10	25
Performance	19	60	Values	10	40
Attitudes	18	50	Consumer	9	37
Behaviour	18	47	Fast fashion	9	23
Management	15	48	Green	9	29
Apparel	12	42	Perceptions	9	25

## Appendix 2. Recurrent abstract terms

**Table:**

Terms	Occurrences	Relevance score	Terms	Occurrences	Relevance score
Study	240	0.2383	Paper	69	1.306
Consumer	162	0.7988	Company	68	20.069
Sustainability	107	0.243	Fashion industry	67	0.4945
Practice	105	12.427	Clothing	66	15.478
Corporate social responsibility	104	28.163	Apparel	63	0.6268
Fashion	87	0.4291	Circular economy	61	25.463
Attitude	83	14.353	Textile	58	20.889
Impact	81	0.1315	Factor	55	0.2291
Value	78	0.3641	Influence	49	0.634
Research	75	0.2997	Role	49	0.1475
